# Supplementary material for: The role of the human hippocampus in decision-making under uncertainty
Source: Nat Hum Behav. 2024 Apr 29;8(7):1366–82. doi: 10.1038/s41562-024-01855-2 (PMC11272595; doi:10.1038/s41562-024-01855-2)
Supplement: Supplementary file 1 — Supplementary Figs. 1–8, Tables 1–25, analyses and discussions. [file 41562_2024_1855_MOESM1_ESM.pdf]

nature human behaviour

Article https://doi.org/10.1038/s41562-024-01855-2

The role of the human hippocampus in decision-making under uncertainty

In the format provided by the authors and unedited

Supplementary Information

Expected error and uncertainty

Objective uncertainty in *Circle Quest* task was quantified as the expected error of localisation (EE). This is equal to the error the ideal agent would obtain on average by placing the blue disc at the best possible location given the information on the screen. For each location λ on the screen the probability that this location is the centre of the hidden circle given the observation o at location σ can be calculated using Bayes' rule as follows:

$$\begin{aligned} p_s(\lambda|o,\sigma) &= \frac{p_s(\lambda).p_s(o,\sigma|\lambda)}{p_s(o,\sigma)} \\ p_{s+1}(\lambda) &= p_s(\lambda|o,\sigma) \end{aligned} \tag{1}$$

With successive sampling, this rule is applied sequentially. Therefore, the posterior probability $p_s(\lambda|o,\sigma)$ becomes the prior probability $p_{s+1}(\lambda)$ from one sample to the next.

Given the rules of the task and that there is no uncertainty regarding the radius of the hidden circle, the likelihood of observing a purple dot (o^+) at a location σ is 1 for locations within one radius distance of σ, and zero otherwise. The opposite is true for the likelihood of observing a white dot (o^-). Thus, the likelihood function can be expressed mathematically as:

$$\begin{cases} p_s(o^+,\sigma|\lambda) = 1 \text{ if } |\lambda-\sigma| \leq r \\ p_s(o^+,\sigma|\lambda) = 0 \text{ if } |\lambda-\sigma| > r \\ p_s(o^-,\sigma|\lambda) = 1 - p_s(o^+,\sigma|\lambda) \end{cases} \tag{2}$$

Where r is the radius of the hidden circle which is fixed.

The probability of the observation o at the sampling location σ is the sum over all possible hidden circle centres λ of the probability of the observation given λ, weighted by the probability of λ to be the hidden circle centre:

$$p_s(o,\sigma) = \sum_{\lambda} p_s(o,\sigma|\lambda).p_s(\lambda) \tag{3}$$

Thus, for every possible circle placement, an expected error can be computed as:

$$EE_s(\lambda) = \sum_i p_s(\lambda_i).|\lambda - \lambda_i| \tag{4}$$

Computational modelling of active information gathering

To further characterise active information sampling performance in Exp. 1, we analysed the behaviour using a well-validated computational model previously implemented in healthy and patient groups[1,2].

The model calculates the expected utility of a sample (EU_s) accounting for economic and hidden cognitive effort costs to return five parameter estimates per participant. The first two parameters represent the weights participants assign to sample costs (w_s) and benefits (w_e). Two parameters describe the cognitive cost function $\eta_c(ISI, \alpha)$ in terms of a penalty for sampling speed (w_{speed}) and efficiency (w_α). The fifth parameter represents an intercept per participant describing their baseline valuation of samples (w_0).

This was formalised quantitatively as follows:

$$\begin{aligned} &EU_s(ISI, \alpha, t_{max}) = EU_{s-1} + p(s|ISI, t_{max}).[w_e.\eta_e.(1-\alpha).(EE_{s-1} - \hat{EE}_\infty) \\ &- w_s.\eta_s^{1+\gamma.s} - \eta_c(ISI, \alpha)] \\ &\text{Previous EU} + \text{Probability of acquiring the sample given the current time} \\ &.\,[\text{Expected information benefit} - \text{Sampling cost} - \text{Cognitive effort cost}] \end{aligned} \tag{5}$$

where η_e is the placement error penalty (1.2 credits/pixel) and t_{max} is the allowed search time per trial (18 seconds). $\hat{EE}_\infty$ is the per-individual information sampling asymptotic limit estimated beforehand to take into consideration inter-participant variations in asymptotic information sampling performance.

Based on previous work[1,2], we used quadratic cognitive cost function as follows:

$$\eta_c(ISI, \alpha) = w_0 + w_{speed} \times \frac{1}{ISI^2} + w_\alpha \times \alpha^2 \tag{6}$$

To obtain the likelihood function, *softmax* function was applied over the 3-dimensional space of EU (EU depends on ISI, α, s) for a given task condition as follows:

$$p_s(stop|ISI, \alpha, t_{max}) = \frac{\exp(EU_s(ISI, \alpha, t_{max}))}{\sum_i \sum_a \sum_t^{t_{max}} \exp(EU_s(i, a, t))} \tag{7}$$

For each individual, model fitting involved findings the parameters that achieved the lowest negative log-likelihood of observing the multivariate distribution of the number of samples acquired (s), inter-sampling interval (ISI) and sampling efficiency (α).

Optimisation of parameters was performed in MATLAB (The MathWorks inc., version 2019a) using Bayesian Adaptive Direct Search (BADS[3]). Further information about this modelling framework is provided in[1,2].

After the exclusion of potential outliers (1 patient with values $> 3SD$), comparing parameter estimates between two groups showed that ALE patients had lower weights assigned to sampling cost compared to controls ($t_{35} = -2.24$, $95\%CI = [-0.077, -0.003]$, $p = 0.0315$, $Cohen's\ d = -0.72$; Figure S1). There was no significant difference between the two groups in any of the other parameters. These results thus represent a computational formalisation of the findings from Exp. 1 suggesting that ALE patients have lower sensitivity to the cost of sampling.

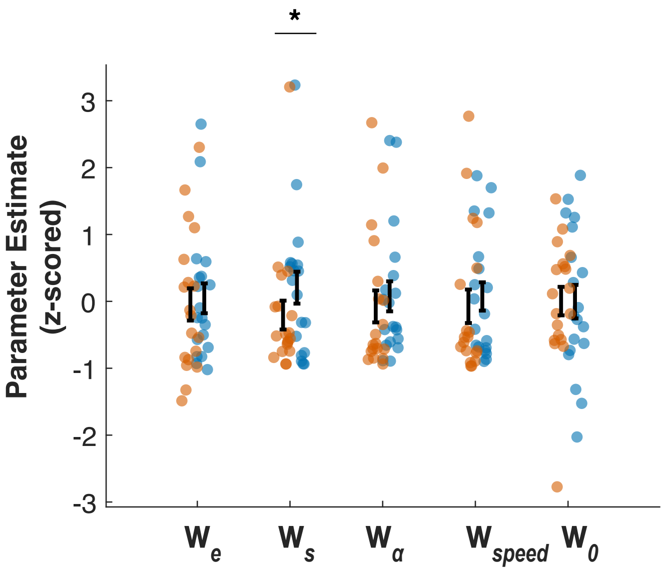

Figure S1: **Computational modelling of active information sampling (Exp. 1).** Compared to healthy matched controls, ALE patients assigned lower economic costs (w_s) to sample acquisition ($t_{35} = -2.24,\ 95\%CI = [-0.077, -0.003],\ p_{uncorr} = 0.031,\ Cohen's\ d = -0.72$, 18 patients and 19 controls). All other model parameters including weights assigned to sample benefit (w_e), efficiency w_α, and speed (w_{speed}) were not significantly different between patients and controls. w_0 captures a subjective fixed cost of sampling that is not explicitly specified in the task (e.g., cost of the motor action). This was not significantly different between the two groups. Error bars show ± SEM.

Decision times

Decision times across the three passive decision making tasks (Exps 2–3) were compared to gain further insights into the cognitive process involved. The informative comparison is mainly between Exp. 2 and Exp. 3 given that they both feature only two attributes (compared to three attributes in Exp. 4) and have an equal number of participants.

No significant difference in reaction time emerged between Exp. 2 and Exp. 3 across all participants ($\beta = 0.128,\ t_{91} = 0.94,\ p = 0.35$, Figure S2 Table S24), and the interaction of group × Exp was also not significant ($\beta = 0.225,\ t_{91} = 1.17,\ p = 0.25$). Comparing reaction time within the ALE group across the two Exps. reveals that ALE patients actually tool longer in Exp. 3 (reward & effort) Compared to Exp. 2 (reward & uncertainty) (Exp. 2: $\mu = 2.04,\ SD = \pm 0.40$, Exp 3: $\mu = 2.40,\ SD = \pm 0.49,\ t_{18} = 2.25,\ p = 0.03$). This could indicate that trials in Exp. 2 require less deliberation for ALE patients, possibly implying the disregard of other values (such as reward) in the presence of uncertainty. This observation aligns with their preferences and active samples (Exp. 1), which exhibit faster sampling rates.

It is noteworthy that Exp. 4, as expected, demonstrated significantly increased decision time ($\beta = 1.18,\ t_{91} = 7.68,\ p < 0.0001$), consistent with the more complex decision-making process involving the consideration of three attributes. This result further supports the argument against rapid responding discussed in the subsection of the Results.

While these findings shed light on task performance difficulty, it is important to acknowledge that reaction times may not entirely negate the presence of a complexity effect. To control for this effect, it might be necessary to conduct novel experiments with a redesigned task, requiring participants to infer uncertainty and effort levels using analogous cues (e.g., levels on a bar). This approach aims to eliminate any additional cognitive effort needed to infer these attributes.

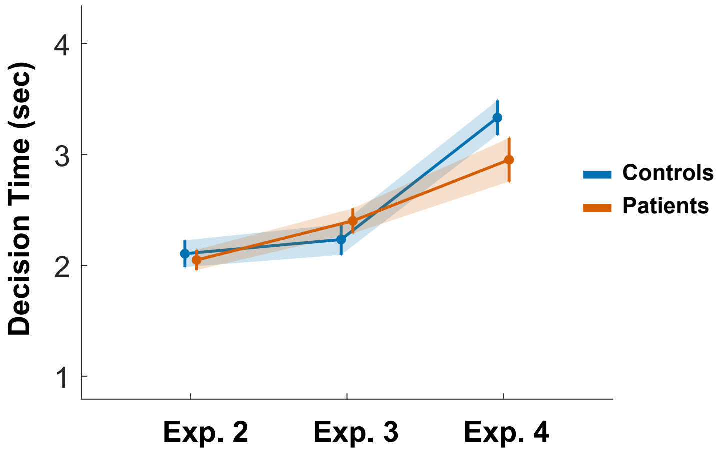

Figure S2: **Decision times measured in seconds (sec) in Exps. 2–4.** Across passive decision tasks (Exps. 2–4), no significant difference was found between ALE patients (N = 19 in Exp. 2 & 3, N = 8 in Exp. 4) and controls (19 in Exp. 2 & 3, N = 12 in Exp. 4). ALE patients made faster decisions in Exp. 2 compared to Exp. 3, indicating less deliberation when making decisions under uncertainty compared to effort-based decision making. Exp. 4 had significantly slower decisions, reflecting the more complex task structure with three decision attributes to consider. Error bars and shading represent ±SEM. For full statistical details see Table S24

Supplementary Figures

Figures S3 to S8

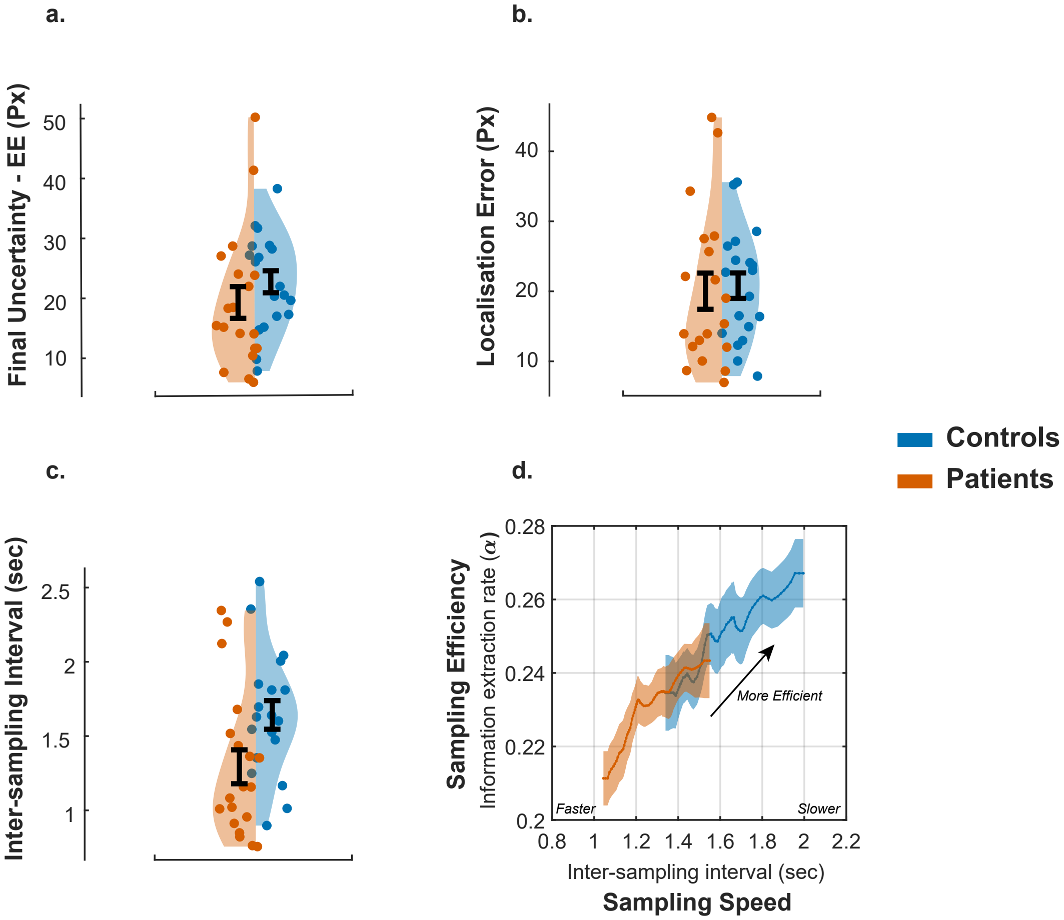

Figure S3: **Active sampling (Exp. 1) – ALE patients commit to decisions at similar uncertainty levels as controls. a.** Final uncertainty is the expected error (*EE*) in pixels (Px) that a participant is likely to obtain at the end of their search. In the experimental condition where ALE patients over-sampled more than controls, there was no significant difference between ALE patients and controls in this measure ($z = -1.60$, $p = 0.108$, $Cliff's\ \delta = -0.30$). **b.** Similarly, the actual error that participants obtained upon localising the circle (distance to hidden circle in pixels) was not significantly different between patients and controls ($z = -0.81$, $p = 0.413$, $Cliff's\ \delta = -0.15$). These two results indicate that ALE patients wasted monetary resources on samples with limited utility (i.e., over-sampled). **c.** In the same condition, ALE patients gathered information at a significantly faster rate than controls ($z = -2.53$, $p = 0.011$, $Cliff's\ \delta = -0.48$). **d.** Sampling behaviour in ALE patients and controls was characterised by a speed-efficiency trade-off whereby faster sampling rates (shorter ISI) were associated with lower sampling efficiency (smaller α). The figure shows this trade-off for the same condition in which patients over-sampled more than controls, demonstrating that ALE patients were also both faster and less efficient than controls. Error bars in a.-c., and shading in d. show ± SEM. Data represent 19 patients and 19 controls. See Tables S4, S6 & S7 for additional statistical details.

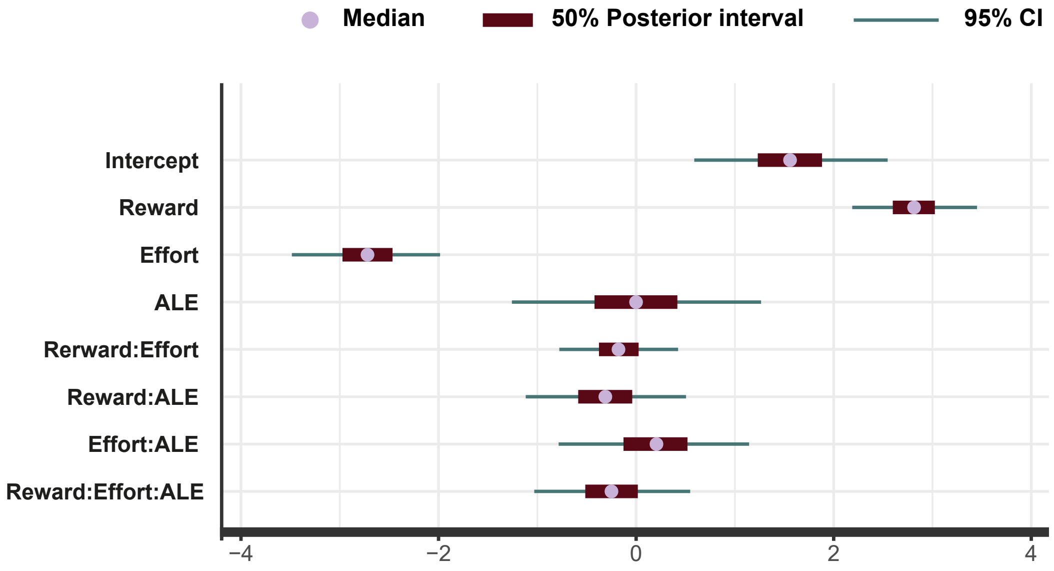

Figure S4: **Baysian mixed-effects model.** The purple dots show the median of the posterior distributed with 95% credible intervals (thin green line) and 50% posterior interval (thick dark red lines). Model was specified as follows:choice $\sim$ 1 + group*Reward + group*Effort + Reward*Effort + group:Reward:Effort + (1 + reward*Effort |participant). N = 19 for each group.

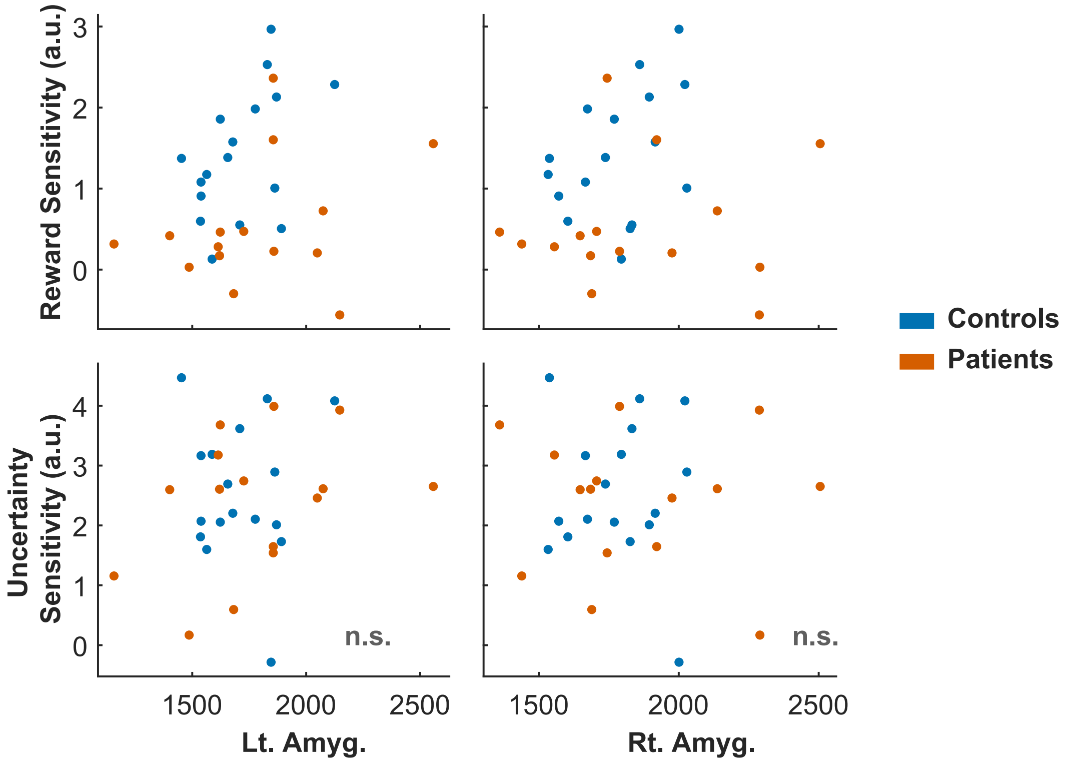

Figure S5: **Amygdala as control region.** No significant correlation was detected between amygdala volume and sensitivity to reward or uncertainty (Robust regression $p > 0.20$ for all correlations).

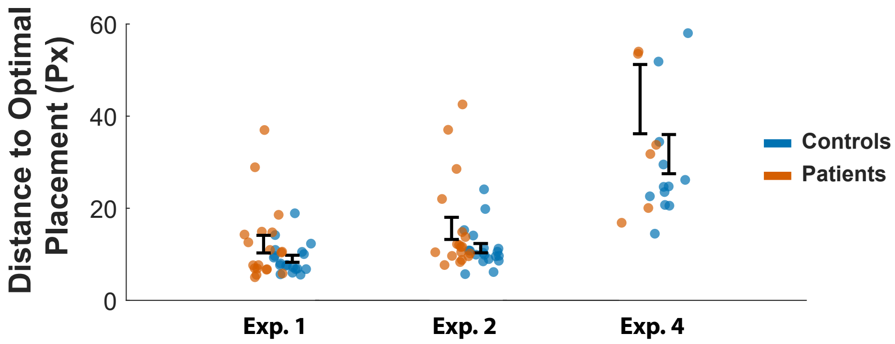

Figure S6: **Intact localisation performance.** Distance to optimal placement is the distance between the centre of the blue disc and the best localisation given the configuration of the dots on display. Across the three versions of *Circle Quest* (Exps. 1, 2 & 4), there was no significant difference between ALE patients and controls in this measure, indicating intact localisation performance. Error bars show ± SEM. In Exps. 1 & 2, N = 19 for both groups. In Exp. 4, N = 12 for controls and 8 for ALE patients.

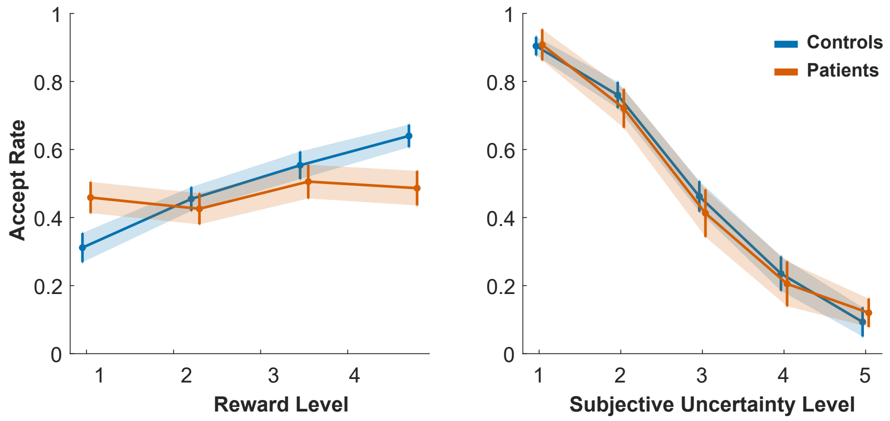

Figure S7: **Passive choices as a function of reward and subjective uncertainty estimates**. There is no change in choice performance results when subjective estimates of uncertainty are used instead of expected error (EE) in the analysis. ALE patients (N = 19) demonstrate lower sensitivity to reward and intact sensitivity to uncertainty when compared to healthy controls (N = 19). Reward levels 1-4 correspond to the number of credits on display (R: 40, 65, 90, 115 credits). Subjective uncertainty levels were calculated by binning sign-flipped z-scored confidence ratings into five bins. level five describes the lowest level of subjective uncertainty estimate. Error bars and shading represent ±SEM. For statistical details see Table S18.

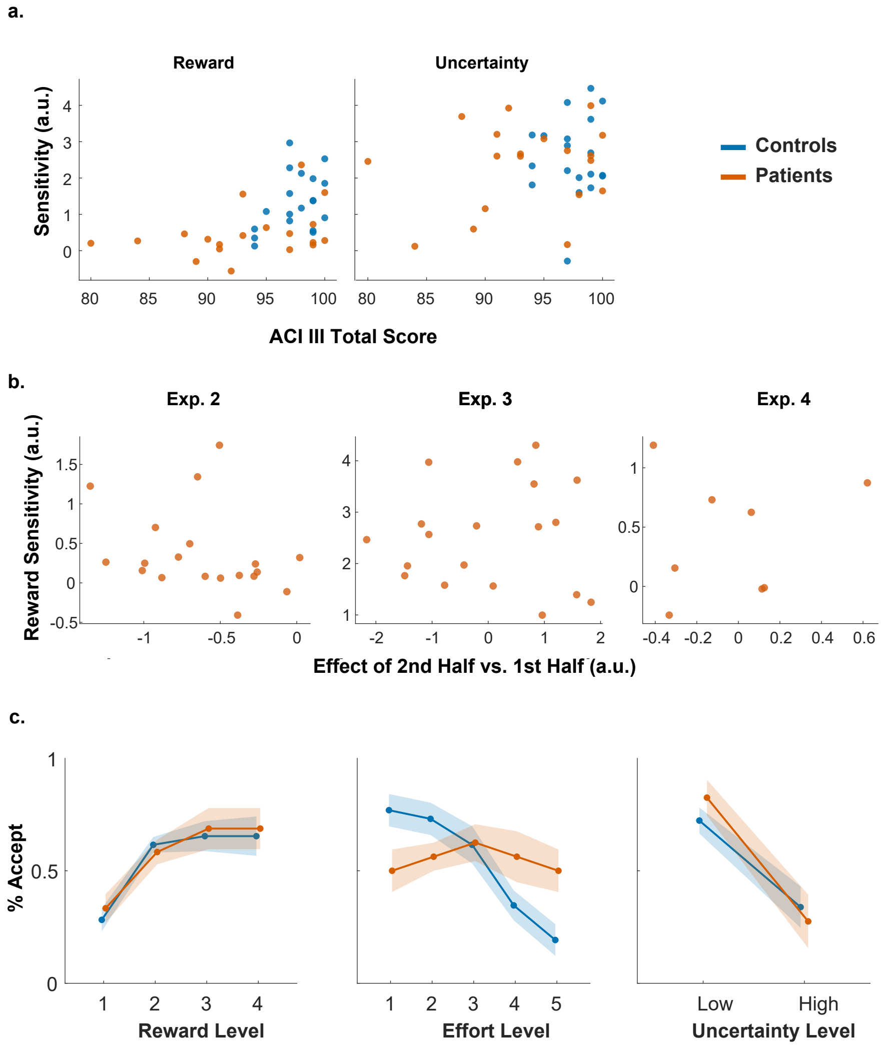

Figure S8: **Minimal effect of cognitive deficit and memory decay on performance**. **a.** There was no significant correlation between cognitive scores indexed by ACE-III scores and sensitivity to either reward or uncertainty in Exp. 2, indicating that the difference between ALE patients and controls is likely related to cognitive dysfunction. **b.** Whether trials were played in the second half of the experiment compared to the first half did not have a significant effect on reward sensitivity, suggesting minimal presence of memory decay that could influence behaviour or result in random responding. **c.** Catch trials in Exp. 4 show that ALE patients had intact sensitivity to uncertainty (right panel) and blunted sensitivity to effort (middle panel), pointing against random responding during the task, and replicating results from the main task trails. Reward sensitivity (left panel) is intact in these trials but this should be interpreted with caution as reward represented in a balanced design in these catch trials. Shaded area around the lines indicate $\pm SEM$. For statistical details see Tables S20, S22 & S23.

Supplementary Tables

Tables S1 to S25

	Controls		Patients		
**Count (M/F)**	19 (13/6)		19 (13/6)		
**Variable**	**Mean**	**SD**	**Mean**	**SD**	**p-value**
**Age**	61.16	11.71	60.00	11.36	0.76
**ACE-III**	97.52	2.03	93.42	5.64	0.005
**DS**	18.05	3.49	19.79	5.00	0.21
**AMI**	1.18	0.40	1.25	0.49	0.65
**FSS**	3.06	1.12	3.36	1.86	0.59
**BDI-II**	5.9	5.26	10.95	10.15	0.06
**SHAPS**	18.84	4.56	21.58	4.85	0.08
**Lt. Hipp Volume***	3495.06	199.41	3357.71	722.71	0.457
**Rt. Hipp Volume***	3662.99	167.06	3288.50	675.58	0.034

Table S1: **Demographics.** ACE-III: Addenbrooke's Cognitive Examination. DS: Digit Span. AMI: Apathy Motivation Index. FSS: Fatigue Severity Scale. BDI-II: Beck Depression Inventory. SHAPS: Snaith-Hamilton Pleasure Scale. Hipp: Adjusted Hippocampal Volumes.* 15 patients and 17 controls. Statistical testing was performed with a two-sample t-test if data fulfilled parametric assumptions or a Wilcoxon rank-sum test if assumptions were violated.

Code	Age	Gender	Abs	Lt. Hipp.		Rt. Hipp.		Years Since Diagnosis	Years Since First Symptom
				**Raw Volume (adjusted)**	**Percentile**	**Raw Volume (adjusted)**	**Percentile**		
1	53	F	LGI1	3559.64 (3710.42)	29	3547.33 (3725.45)	21	3.38	5.27
2	47	F	LGI1	3157.67 (3111.00)	6[c]	2717.64 (2662.51)	<2.5[c]	3.44	3.77
3	59	F	LGI1	2332.83 (2313.53)	<2.5	2255.53 (2232.73)	<2.5	3.49	9.74
4	63	F	LGI1	2860.65 (2941.60)	<2.5	3744.13 (3839.76)	43	2.59	2.92
5	72	F	LGI1	3170.72 (3204.72)	17[c]	2495.43 (2535.59)	<2.5[c]	7.67	7.67
6	64	M	LGI1	-	-	-	-	4.66	4.82
7	55	M	LGI1	4835.20 (4969.31)	97	4125.12 (4283.55)	46	2.56	2.64
8	53	M	LGI1	3663.60 (3710.05)	19	3754.24 (3809.11)	17	2.36	3.21
9	66	M	LGI1	4109.11 (4115.25)	72	4341.71 (4348.97)	80	1.18	1.77
10	65	M	LGI1	3488.01 (3240.14)	18	3379.20 (3086.38)	9	3.27	4.11
11	72	M	LGI1	2973.83 (2811.09)	5	2905.04 (2712.78)	3	1.82	1.9
12	26	M	LGI1	-	-	-	-	0.96	0.97
13	68	M	CASPR2	3050.27 (2970.48)	4	3364.09 (3269.84)	10	1.03	1.08
14	77	M	CASPR2	-	-	-	-	10.52	10.52
15	65	M	CASPR2	3455.68 (3311.09)	16	3411.77 (3240.95)	10	5.29	6.87
16	67	M	CASPR2	4118.84 (4163.74)	74	3942.82 (3995.87)	48	4.30	5.47
17	58	F	LGI1/CASPR2	3670.25 (3590.67)	41	3073.41 (2979.41)	4	3.26	3.83
18	58	M	LGI1/CASPR2	-	-	-	-	7.16	7.16
19	52	M	Seronegative	2370.64 (2228.48)	<2.5[c]	2752.98 (2585.05)	<2.5[c]	1.99	2.78

Table S2: **Patients Characteristics**. Abs: Autoantibodies. Lt. Hipp.: Left Hippocampus. Rt. Hipp.: Right Hippocampus. Hippocampal volumes were adjusted for intra-cranial volumes. Percentile is determined by plotting raw hippocampal volumes against normative brain volumes from UK biobank data[4]. [c]: Describes percentiles outside the age range of the UK biobank nomograms. Percentiles according to the closest age value within the UK biobank range was used instead.

Code	Clinical Profile on Presentation	Clinical profile in Chronic Phase	Acute Management	Medications in Chronic Phase
1	Memory deficits, irritability, falls	Seizures	IVIG (x3), PLEX (x1), Steroids	Steroids (low dose), Levetiracetam, Lamotrigine
2	Seizures (numbness and weakness left hand), anxiety, fatigue	Memory deficits, emotional liability, abnormal sensation left hand	na	Steroids (low dose), Mycophenolate, Carbamazepine
3	Seizures, memory deficits	Seizures	Steroids, azathioprine (briefly), methotrexate, carbamazepine, lacosamide, PLEX	Carbamazepine, Lacosamide
4	FBDS, memory deficits, auditory hallucinations, anxiety, falls	Poor concentration, fatigue, apathy	Steroids, Azathioprine, PLEX, levetiracetam, mycophenolate, Rituximab	Steroids (low dose), Mycophenolate, Levetiracetam
5	Neurocardiac syndrome (tachi-bradycardia), seizures (thermal and sensory sensations and one tonic-clonic), increased daytime sleepiness, headache, fatigue, brain fog	Anxiety, abdominal sensations	Steroids, levetiracetam	Levetiracetam
6	Behavioural changes, nocturnal seizures	No symptoms	na	Carbamazepine
7	FBDS, memory deficits	Memory deficits	Steroids, Clobazam, PLEX	Steroids (low dose), Pregabalin
8	Seizures (including hysterical laughing), hypersomnia, anxiety, memory deficits, cough, breathlessness	No symptoms	IVIG, Steroids, lacosamide	Steroid (low dose), Mycophenolate, Lacosamide
9	FBDS	No symptoms	Steroids, Lamotrigine	Steroids (lower dose), Lamotrigine
10	Seizures, behavioural change (apathy), memory deficits	Apathy, memory deficits	na	None
11	Seizures (tingling, lateralized weakness), memory deficits	Memory deficits	Steroids, Lamotrigine	Steroids (low dose), Lamotrigine
12	FBDS, memory deficits	No symptoms	Steroids, PLEX, Levetiracetam	Steroids (low dose), Levetiracetam
13	Seizures, memory deficits, emotional liability, sleep cycle inversion	Memory and concentration deficits, headaches, leg pain	Steroids, levetiracetam, PLEX, Pregabalin	Steroids (low dose), Levetiracetam, Pregabalin
14	Morvan's syndrome, speech and balance problems	Neuropathic pain, problems with balance, muscle twitching, memory deficits	Mycophenolate, Steroids, Pregabalin	Steroids (low dose), Pregabalin, Sertraline
15	Seizures, memory deficits	Memory deficits	Lamotrigine, Steroids	Lamotrigine
16	Seizures, behavioural change, hallucinations	Memory deficits, fatigue	Lacosamide	Lacosamide
17	Lower limbs pain, insomnia, muscle twitching, sweating, abdominal bloating, Morvan's syndrome, rash, confusion, hallucinations, seizures	Pain and numbness in lower limbs, muscle twitching, fatigue	Steroids, IVIG, PLEX, Cyclophosphamide, Phenytoin, Pregabalin, Mirtazapine	Steroids (low dose), Phenytoin, Pregabalin
18	Memory deficits	Memory deficits	Cyclophosphamide, Phenytoin	Steroids (low dose), Levetiracetam
19	Seizure, fatigue, apathy, delusion, irritability	Seizures, Memory deficits, anxiety, verbally aggressive	Steroids, Lacosamide, Levetiracetam, Citalopram	Lacosamide, Levetiracetam, Citalopram

Table S3: **Patients Clinical Profiles.** FBDS: Faciobrachial dystonic seizures. IVIG: intravenous immunoglobulin. PLEX: Plasma exchange. na: data not available from patients local records.

	S	score	EE	Error	ISI
(Intercept)	$\beta = +2.18$	$\beta = +65.7$	$\beta = +20.3$	$\beta = +2.06$	$\beta = +1.5$
	$SE = 0.0817$	$SE = 1.39$	$SE = 2.12$	$SE = 0.0873$	$SE = 0.0902$
	$t_{2272} = +26.72$	$t_{2272} = +47.32$	$t_{2272} = +9.57$	$t_{2272} = +23.64$	$t_{2272} = +16.67$
	p<0.0001	***p<0.0001***	***p<0.0001***	***p<0.0001***	***p<0.0001***
ALE	$\beta = +0.153$	$\beta = -7.93$	$\beta = -0.683$	$\beta = +0.162$	$\beta = -0.211$
	$SE = 0.116$	$SE = 1.96$	$SE = 2.99$	$SE = 0.123$	$SE = 0.128$
	$t_{2272} = +1.32$	$t_{2272} = -4.04$	$t_{2272} = -0.23$	$t_{2272} = +1.32$	$t_{2272} = -1.65$
	$p = 0.19$	***p<0.0001***	$p = 0.82$	$p = 0.19$	$p = 0.10$
ALE:R_0	$\beta = +0.0247$	$\beta = -1.66$	$\beta = -0.952$	$\beta = -0.0265$	$\beta = -0.0393$
	$SE = 0.0251$	$SE = 1.06$	$SE = 0.819$	$SE = 0.0376$	$SE = 0.027$
	$t_{2272} = +0.98$	$t_{2272} = -1.56$	$t_{2272} = -1.16$	$t_{2272} = -0.70$	$t_{2272} = -1.45$
	$p = 0.33$	$p = 0.12$	$p = 0.25$	$p = 0.48$	$p = 0.15$
ALE:η_s	$\beta = +0.0318$	$\beta = -3.66$	$\beta = -0.924$	$\beta = -0.0277$	$\beta = -0.0719$
	$SE = 0.0247$	$SE = 1.84$	$SE = 0.665$	$SE = 0.0318$	$SE = 0.0337$
	$t_{2272} = +1.29$	$t_{2272} = -2.00$	$t_{2272} = -1.39$	$t_{2272} = -0.87$	$t_{2272} = -2.14$
	$p = 0.20$	***p = 0.046***	$p = 0.16$	$p = 0.38$	***p = 0.033***
ALE:η_s:R_0	$\beta = +0.0542$	$\beta = -0.403$	$\beta = -0.881$	$\beta = -0.0463$	$\beta = -0.0273$
	$SE = 0.0202$	$SE = 1.04$	$SE = 0.602$	$SE = 0.0302$	$SE = 0.0231$
	$t_{2272} = +2.68$	$t_{2272} = -0.39$	$t_{2272} = -1.46$	$t_{2272} = -1.53$	$t_{2272} = -1.18$
	p = 0.0074	$p = 0.70$	$p = 0.14$	$p = 0.13$	$p = 0.24$
R_0	$\beta = +0.0231$	$\beta = +17.6$	$\beta = -0.169$	$\beta = +0.0104$	$\beta = +0.00487$
	$SE = 0.018$	$SE = 0.751$	$SE = 0.579$	$SE = 0.0266$	$SE = 0.0191$
	$t_{2272} = +1.29$	$t_{2272} = +23.45$	$t_{2272} = -0.29$	$t_{2272} = +0.39$	$t_{2272} = +0.25$
	$p = 0.20$	***p<0.0001***	$p = 0.77$	$p = 0.70$	$p = 0.80$
η_s	$\beta = -0.111$	$\beta = -18.7$	$\beta = +2.07$	$\beta = +0.0919$	$\beta = +0.12$
	$SE = 0.0177$	$SE = 1.3$	$SE = 0.47$	$SE = 0.0225$	$SE = 0.0238$
	$t_{2272} = -6.25$	$t_{2272} = -14.41$	$t_{2272} = +4.40$	$t_{2272} = +4.09$	$t_{2272} = +5.04$
	p<0.0001	***p<0.0001***	***p<0.0001***	***p<0.0001***	***p<0.0001***
η_s:R_0	$\beta = -0.0301$	$\beta = -0.654$	$\beta = +0.477$	$\beta = +0.0211$	$\beta = +0.0135$
	$SE = 0.0145$	$SE = 0.732$	$SE = 0.426$	$SE = 0.0213$	$SE = 0.0164$
	$t_{2272} = -2.07$	$t_{2272} = -0.89$	$t_{2272} = +1.12$	$t_{2272} = +0.99$	$t_{2272} = +0.83$
	p = 0.038	$p = 0.37$	$p = 0.26$	$p = 0.32$	$p = 0.41$
$adj - R^2$	0.79	0.71	0.58	0.27	0.71
N_{obs}	2280	2280	2280	2280	2280
AIC	295.84	19913.90	16521.03	4656.47	1163.69

Table S4: **Active Search (Exp. 1) – Generalised mixed-effects models of the effect of the group (ALE) on performance compared to controls**. Models were specified as follows: Predicted variable $\sim$ 1 + group*η_s + group*R_0 + η_s*R_0 + group:η_s:R_0 + (1 + η_s*R_0 |participant). Control group was set as reference group. S: Raw number of samples. EE: Expected Error (Uncertainty). Error: Distance to hidden circle. ISI: Inter-Sampling Interval. η_s : Sampling Cost. R_0: Initial Reward Reserve.

Condition (R/η_s)	Controls	ALE
Low/Low	$\beta = -2.24$<br>$SE = 0.715$<br>$t_{1136} = -3.14$<br>***p = 0.0018***	$\beta = -1.6$<br>$SE = 1.23$<br>$t_{1136} = -1.30$<br>$p = 0.19$
$Low/High$	$\beta = +1.97$<br>$SE = 0.543$<br>$t_{1136} = +3.63$<br>***p = 0.00029***	$\beta = +3.14$<br>$SE = 0.81$<br>$t_{1136} = +3.88$<br>***p = 0.00011***
$High/Low$	$\beta = -1.32$<br>$SE = 0.689$<br>$t_{1136} = -1.92$<br>$p = 0.06$	$\beta = -0.277$<br>$SE = 1.34$<br>$t_{1136} = -0.21$<br>$p = 0.84$
$High/High$	$\beta = +1.9$<br>$SE = 0.625$<br>$t_{1136} = +3.04$<br>***p = 0.0024***	$\beta = +4.74$<br>$SE = 1.05$<br>$t_{1136} = +4.52$<br>***p<0.0001***
$adj - R^2$	0.86	0.84
N_{obs}	1140	1140
AIC	4273.50	5545.49

Table S5: **Active Search (Exp. 1) – Generalised mixed-effects models investigating deviation from optimal number of samples.**. Models were specified as follows: $Deviation \sim Condition$ + ($Condition$ |participant). R : Initial reward reserve. η_s : Sampling cost.

	Controls	ALE
(Intercept)	$\beta = -1.67$<br>$SE = 0.0721$<br>$t_{1138} = -23.12$<br>***p<0.0001***	$\beta = -1.9$<br>$SE = 0.0881$<br>$t_{1138} = -21.57$<br>***p<0.0001***
ISI	$\beta = +0.137$<br>$SE = 0.0463$<br>$t_{1138} = +2.96$<br>***p = 0.0032***	$\beta = +0.26$<br>$SE = 0.054$<br>$t_{1138} = +4.82$<br>***p<0.0001***
$adj - R^2$	0.22	0.27
N_{obs}	1140	1140
BIC	570.28	858.72

Table S6: **Active Search (Exp. 1) – Generalised mixed-effects models investigating the relationship between inter-sampling interval and information extraction rate.** Model was specified as follows: $\alpha \sim$ 1 + ISI + (1 |trial) + (1 + ISI |condition) + (1 + ISI |participant)

	**Exp. 1**
(Intercept)	$\beta = +0.244$<br>$SE = 0.00934$<br>$t_{566} = +26.11$<br>***p<0.0001***
ALE	$\beta = -0.026$<br>$SE = 0.0131$<br>$t_{566} = -1.98$<br>***p = 0.048***
ALE:ISI	$\beta = -0.00165$<br>$SE = 0.0128$<br>$t_{566} = -0.13$<br>$p = 0.90$
ISI	$\beta = +0.0122$<br>$SE = 0.00905$<br>$t_{566} = +1.35$<br>$p = 0.18$
$adj - R^2$	0.25
N_{obs}	570
AIC	-1256.45

Table S7: **Active Search (Exp. 1) – Generalised mixed-effects model investigating the effect of ALE on efficiency (α) in the condition with high sampling cost and high initial reward reserve, i.e., the condition where ALE patients over-sampled more than controls.** Model was specified as follows. : $\alpha \sim$ 1 + group*ISI + (1 |trial) + (1 + ISI |participant).

	**Exp. 2**
(Intercept)	$\beta = -0.423$<br>$SE = 0.323$<br>$t_{3748} = -1.31$<br>$p = 0.19$
ALE	$\beta = -0.402$<br>$SE = 0.457$<br>$t_{3748} = -0.88$<br>$p = 0.38$
ALE:EE	$\beta = +0.336$<br>$SE = 0.442$<br>$t_{3748} = +0.76$<br>$p = 0.45$
ALE:R	$\beta = -0.983$<br>$SE = 0.275$<br>$t_{3748} = -3.58$<br>***p = 0.00035***
ALE:R:EE	$\beta = +0.162$<br>$SE = 0.17$<br>$t_{3748} = +0.95$<br>$p = 0.34$
EE	$\beta = -2.73$<br>$SE = 0.313$<br>$t_{3748} = -8.72$<br>***p<0.0001***
R	$\beta = +1.41$<br>$SE = 0.198$<br>$t_{3748} = +7.16$<br>***p<0.0001***
R:EE	$\beta = +0.0659$<br>$SE = 0.125$<br>$t_{3748} = +0.53$<br>$p = 0.60$
$adj - R^2$	0.93
N_{obs}	3756
AIC	2938.44

Table S8: **Passive choices (Exp. 2) – Generalised mixed-effects model examining effect of reward and uncertainty on choices as well as differences between ALE group and controls.** Model was specified as follows: choice $\sim$ 1 + group*R + group*EE + R*EE + group:R:EE + (1 + R*EE |participant). Control group was set as the reference group. EE: Expected Error. R: Reward. ALE: Autoimmune Limbic Encephalitis.

	Exp. 2 with outlier removed
(Intercept)	$\beta = -0.59$<br>$SE = 0.315$<br>$t_{3648} = -1.87$<br>$p = 0.06$
LE	$\beta = -0.226$<br>$SE = 0.439$<br>$t_{3648} = -0.52$<br>$p = 0.61$
LE:EE	$\beta = +0.529$<br>$SE = 0.412$<br>$t_{3648} = +1.28$<br>$p = 0.20$
LE:R	$\beta = -0.868$<br>$SE = 0.259$<br>$t_{3648} = -3.35$<br>***p = 0.00081***
LE:R:EE	$\beta = +0.18$<br>$SE = 0.173$<br>$t_{3648} = +1.04$<br>$p = 0.30$
EE	$\beta = -2.9$<br>$SE = 0.298$<br>$t_{3648} = -9.73$<br>***p<0.0001***
R	$\beta = +1.31$<br>$SE = 0.189$<br>$t_{3648} = +6.97$<br>***p<0.0001***
R:EE	$\beta = +0.0843$<br>$SE = 0.128$<br>$t_{3648} = +0.66$<br>$p = 0.51$
$adj - R^2$	0.91
N_{obs}	3656
AIC	2879.44

Table S9: **Passive choices (Exp. 2) – Generalised mixed-effects model of the effect of the group (ALE vs. Controls) on choices with one outlier removed from the control group.** Model was specified as follows: choice $\sim$ 1 + group*R + group*EE + R*EE + group:R:EE + (1 + R*EE |participant). EE : Expected Error. R :Reward.

	**(Exp. 3)**
(Intercept)	$\beta = +1.96$<br>$SE = 0.442$<br>$t_{4739} = +4.43$<br>***p<0.0001***
ALE	$\beta = -0.137$<br>$SE = 0.622$<br>$t_{4739} = -0.22$<br>$p = 0.83$
ALE:Effort	$\beta = +0.306$<br>$SE = 0.467$<br>$t_{4739} = +0.66$<br>$p = 0.51$
ALE:Reward	$\beta = -0.48$<br>$SE = 0.37$<br>$t_{4739} = -1.30$<br>$p = 0.19$
ALE:Reward:Effort	$\beta = -0.22$<br>$SE = 0.408$<br>$t_{4739} = -0.54$<br>$p = 0.59$
Effort	$\beta = -2.82$<br>$SE = 0.334$<br>$t_{4739} = -8.45$<br>***p<0.0001***
Reward	$\beta = +2.97$<br>$SE = 0.267$<br>$t_{4739} = +11.12$<br>***p<0.0001***
Reward:Effort	$\beta = -0.281$<br>$SE = 0.292$<br>$t_{4739} = -0.96$<br>$p = 0.34$
$adj - R^2$	0.97
N_{obs}	4747
AIC	2775.57

Table S10: **Effort-based decision making (Exp. 3)– Generalised mixed-effects models examining effect of reward and effort on choices as well as differences between ALE group and controls.** Models were specified as follows. Effort-based choices: choice $\sim$ 1 + group*Reward + group*Effort + Reward*Effort + group:Reward:Effort + (1 + Reward*Effort $|$participant). Controls group was set as the reference group. ALE: ALE: Autoimmune Limbic Encephalitis group.

Parameter	Rhat	n_eff	mean	sd	2.5%	50%	97.5%
Intercept	1.0	2906	1.6	0.5	0.6	1.6	2.5
Reward	1.0	6786	2.8	0.3	2.2	2.8	3.5
Effort	1.0	5174	-2.7	0.4	-3.5	-2.7	-2.0
Reward:Effort	1.0	7592	-0.2	0.3	-0.8	-0.2	0.4
ALE	1.0	3097	0.0	0.6	-1.3	-0.0	1.3
Reward:ALE	1.0	6647	-0.3	0.4	-1.1	-0.3	0.5
Effort:ALE	1.0	4945	0.2	0.5	-0.8	0.2	1.1
Reward:Effort:ALE	1.0	7563	-0.2	0.4	-1.0	-0.2	0.5

Table S11: **Baysian mixed-effects modelling of effort-based choices data (Exp. 3) – Posterior summary statistics**. Model was specified as follows: choice $\sim$ 1 + group*R + group*EE + R*EE + group:R:EE + (1 + R*EE |participant). To improve convergence and guard against over-fitting, mildly informative conservative priors were specified.

	**Exps. 2 & 3**
(Intercept)	$\beta = +0.0983$<br>$SE = 0.141$<br>$t_{8495} = +0.70$<br>$p = 0.49$
Group	$\beta = -0.235$<br>$SE = 0.202$<br>$t_{8495} = -1.17$<br>$p = 0.24$
Group:Task	$\beta = +0.0471$<br>$SE = 0.102$<br>$t_{8495} = +0.46$<br>$p = 0.65$
Group:Task:Reward	$\beta = +0.467$<br>$SE = 0.113$<br>$t_{8495} = +4.15$<br>***p<0.0001***
Group:Reward	$\beta = -0.569$<br>$SE = 0.159$<br>$t_{8495} = -3.58$<br>***p = 0.00035***
Task	$\beta = +0.534$<br>$SE = 0.0737$<br>$t_{8495} = +7.25$<br>***p<0.0001***
Task:Reward	$\beta = +0.544$<br>$SE = 0.0815$<br>$t_{8495} = +6.68$<br>***p<0.0001***
Reward	$\beta = +0.665$<br>$SE = 0.112$<br>$t_{8495} = +5.94$<br>***p<0.0001***
$adj - R^2$	0.39
N_{obs}	8503
AIC	9824.54

Table S12: **Generalised mixed-effects model examining the effect of Group (ALE) and Task on reward sensitivity in Exps. 2 & 3.** Models were specified as follows. Exps. 2 & 3: choice $\sim$ 1 + Group*Task + Group*Reward + Task*Reward + Group:Task:Reward + (1 + Reward |Participant) + (1 + Reward |Task).

	**Exps. 2 & 3 (ALE only)**
(Intercept)	$\beta = -0.137$
	$SE = 0.144$
	$t_{4224} = -0.95$
	$p = 0.34$
Task	$\beta = +0.582$
	$SE = 0.0709$
	$t_{4224} = +8.20$
	p<0.0001
Task:Reward	$\beta = +1.01$
	$SE = 0.0778$
	$t_{4224} = +13.03$
	p<0.0001
Reward	$\beta = +0.0975$
	$SE = 0.12$
	$t_{4224} = +0.81$
	$p = 0.42$
$adj - R^2$	0.37
N_{obs}	4228
AIC	4973.27

Table S13: **Generalised mixed-effects model examining the effect of task on reward sensitivity in ALE patients.** Models were specified as follows: choice $\sim$ 1 + Task*Reward + (1 + Reward |Participant) + (1 + Reward |Task).

	Exp. 4
(Intercept)	$\beta = +2.23$<br>$SE = 0.621$<br>$t_{4184} = +3.58$<br>***p = 0.00034***
LE	$\beta = -1.04$<br>$SE = 0.996$<br>$t_{4184} = -1.05$<br>$p = 0.29$
ALE:Uncertainty	$\beta = +0.898$<br>$SE = 0.896$<br>$t_{4184} = +1.00$<br>$p = 0.32$
ALE:Uncertainty:Effort	$\beta = -0.541$<br>$SE = 0.275$<br>$t_{4184} = -1.97$<br>***p = 0.049***
ALE:Uncertainty:Reward	$\beta = +0.684$<br>$SE = 0.288$<br>$t_{4184} = +2.37$<br>***p = 0.018***
ALE:Uncertainty:Reward:Effort	$\beta = -0.168$<br>$SE = 0.244$<br>$t_{4184} = -0.69$<br>$p = 0.49$
ALE:Effort	$\beta = +2.06$<br>$SE = 0.554$<br>$t_{4184} = +3.71$<br>***p = 0.00021***
ALE:Reward	$\beta = -1.84$<br>$SE = 0.583$<br>$t_{4184} = -3.16$<br>***p = 0.0016***
ALE:Reward:Effort	$\beta = +0.115$<br>$SE = 0.194$<br>$t_{4184} = +0.59$<br>$p = 0.55$
Uncertainty	$\beta = -1.83$<br>$SE = 0.557$<br>$t_{4184} = -3.29$<br>***p = 0.001***
Uncertainty:Effort	$\beta = +0.558$<br>$SE = 0.191$<br>$t_{4184} = +2.92$<br>***p = 0.0035***
Uncertainty:Reward	$\beta = -0.852$<br>$SE = 0.196$<br>$t_{4184} = -4.35$<br>***p<0.0001***
Uncertainty:Reward:Effort	$\beta = +0.12$<br>$SE = 0.167$<br>$t_{4184} = +0.72$<br>$p = 0.47$
Effort	$\beta = -2.38$<br>$SE = 0.355$<br>$t_{4184} = -6.70$<br>***p<0.0001***
Reward	$\beta = +2.35$<br>$SE = 0.371$<br>$t_{4184} = +6.33$<br>***p<0.0001***
Reward:Effort	$\beta = +0.0131$<br>$SE = 0.133$<br>$t_{4184} = +0.10$<br>$p = 0.92$
$adj - R^2$	0.98
N_{obs}	4200
AIC	3340.31

Table S14: **Generalised mixed-effects model of the effect of the group (LE vs. controls) effort-based decisions under uncertainty (Exp. 4).** Models were specified as follows. EBDM under uncertainty: choice ∼ 1 + group*Uncertainty + group*Reward + Uncertainty*Reward + group*Effort + Uncertainty*Effort + Reward*Effort + group:Uncertainty:Reward + group:Uncertainty:Effort + group:Reward:Effort + Uncertainty:Reward:Effort + group:Uncertainty:Reward:Effort + (1 + Uncertainty*Reward + Uncertainty*Effort + Reward*Effort + Uncertainty:Reward:Effort |participant).

Cluster	Main regions invovled	Voxels	MAX X (mm)	MAX Y (mm)	MAX Z (mm)
1	Right Limbic (Hippocampus, Para-hippocampus, Amygdala)	199	22	-32	-14
2	Thalamus (Bilateral)	120	2	-14	-2
3	Right Temporal Lobe (STG, MTG, AG)	65	68	-40	6

Table S15: **VBM analysis controls vs. patients.** ALE patients had lower grey matter volumes compared to controls in three main clusters (1 –3). The largest difference was seen in the limbic region which includes mainly hippocampal regions. STG: Superior Temporal Gyrus. MTG: Middle Temporal Gyrus. AG: Angular Gyrus.

	**Exps. 1 & 2**	**Exp. 4**
(Intercept)	$\beta = -0.00398$	$\beta = -0.0106$
	$SE = 0.0903$	$SE = 0.174$
	$t_{3752} = -0.04$	$t_{2306} = -0.06$
	$p = 0.96$	$p = 0.95$
EE	$\beta = +0.647$	$\beta = +0.476$
	$SE = 0.0502$	$SE = 0.0873$
	$t_{3752} = +12.88$	$t_{2306} = +5.45$
	p<0.0001	***p<0.0001***
ALE	$\beta = -0.009$	$\beta = +0.028$
	$SE = 0.127$	$SE = 0.281$
	$t_{3752} = -0.07$	$t_{2306} = +0.10$
	$p = 0.94$	$p = 0.92$
ALE:EE	$\beta = -0.0546$	$\beta = -0.0226$
	$SE = 0.0711$	$SE = 0.141$
	$t_{3752} = -0.77$	$t_{2306} = -0.16$
	$p = 0.44$	$p = 0.87$
$adj - R^2$	0.58	0.71
N_{obs}	3756	2310
AIC	7645.65	3940.63

Table S16: **Generalised mixed-effects model of the effect of the group (ALE vs. controls) on flexibility of uncertainty estimation.** Models were specified as follows: Uncertainty Score $\sim$ 1 + group*EE + (1 + EE |participant) + (1 |trial).

	Subjective Uncertainty non-z-scored
(Intercept)	$\beta = -0.407$<br>$SE = 0.022$<br>$t_{3752} = -18.54$<br>***p<0.0001***
EE	$\beta = +0.157$<br>$SE = 0.0122$<br>$t_{3752} = +12.88$<br>***p<0.0001***
Group	$\beta = -0.00219$<br>$SE = 0.031$<br>$t_{3752} = -0.07$<br>$p = 0.94$
Group:EE	$\beta = -0.0133$<br>$SE = 0.0173$<br>$t_{3752} = -0.77$<br>$p = 0.44$
$adj - R^2$	0.58
N_{obs}	3756
AIC	-2972.29

Table S17: **Generalised mixed-effects model examining the effect Group (ALE vs. Controls) on non-z-scored values of subjective uncertainty in Exp 2.** Models were specified as follows. Subjective Uncertainty non-z-scored: Uncertainty (non-z-scored) $\sim$ 1 + group*EE + (1 + EE |participant) + (1 |trial).

	Choices with Confidence (Exp. 2)
(Intercept)	$\beta = +0.361$<br>$SE = 0.367$<br>$t_{3748} = +0.98$<br>$p = 0.33$
Group	$\beta = -0.157$<br>$SE = 0.518$<br>$t_{3748} = -0.30$<br>$p = 0.76$
Group:Reward	$\beta = -1.13$<br>$SE = 0.279$<br>$t_{3748} = -4.06$<br>***p<0.0001***
Group:Reward:Confidence	$\beta = -0.305$<br>$SE = 0.22$<br>$t_{3748} = -1.38$<br>$p = 0.17$
Group:Confidence	$\beta = -0.573$<br>$SE = 0.574$<br>$t_{3748} = -1.00$<br>$p = 0.32$
Reward	$\beta = +1.49$<br>$SE = 0.2$<br>$t_{3748} = +7.47$<br>***p<0.0001***
Reward:Confidence	$\beta = +0.228$<br>$SE = 0.165$<br>$t_{3748} = +1.38$<br>$p = 0.17$
Confidence	$\beta = +3.94$<br>$SE = 0.41$<br>$t_{3748} = +9.62$<br>***p<0.0001***
$adj - R^2$	0.98
N_{obs}	3756
AIC	2436.28

Table S18: **Generalised mixed-effects model examining the effect of Group (ALE), Reward and Subjective Confidence on choices (Exp. 2).** Models were specified as follows. Choices with Confidence: choice $\sim$ 1 + Group*Reward + Group*Confidence + Reward*Confidence + Group:Reward:Confidence + (1 + Reward*Confidence |Participant).

	Choice Model Controlled for Metacognitive Differences
(Intercept)	$\beta = -0.457$<br>$SE = 0.339$<br>$t_{3747} = -1.34$<br>$p = 0.18$
Group	$\beta = -0.351$<br>$SE = 0.481$<br>$t_{3747} = -0.73$<br>$p = 0.47$
Group:EE	$\beta = +0.31$<br>$SE = 0.439$<br>$t_{3747} = +0.70$<br>$p = 0.48$
Group:Reward	$\beta = -0.976$<br>$SE = 0.273$<br>$t_{3747} = -3.57$<br>***p = 0.00036***
Group:Reward:EE	$\beta = +0.165$<br>$SE = 0.172$<br>$t_{3747} = +0.96$<br>$p = 0.34$
EE	$\beta = -2.72$<br>$SE = 0.311$<br>$t_{3747} = -8.73$<br>***p<0.0001***
Metacognitive differences	$\beta = +0.276$<br>$SE = 0.153$<br>$t_{3747} = +1.81$<br>$p = 0.07$
Reward	$\beta = +1.4$<br>$SE = 0.196$<br>$t_{3747} = +7.15$<br>***p<0.0001***
Reward:EE	$\beta = +0.0658$<br>$SE = 0.125$<br>$t_{3747} = +0.53$<br>$p = 0.60$
$adj - R^2$	0.93
N_{obs}	3756
AIC	2938.62

Table S19: **Generalised mixed-effects model examining choice behaviour in Exp. 2 while controlling for differences in uncertainty estimation.** Models were specified as follows. Choice Model Controlled for Metacognitive Differences: Choice $\sim$ 1 + Metacognitive differences + Group*Reward + Group*EE + Reward*EE + Group:Reward:EE + (1 + Reward*EE |participant). Metacognitive differences represent the slope of the correlation between subjective and objective uncertainty estimates.

	**Reward Sensitivity**	**Uncertainty Sensitivity**
(Intercept)	$\beta = -5.9$<br>$SE = 7.31$<br>$t_{13} = -0.81$<br>$p = 0.43$	$\beta = +10.8$<br>$SE = 14.1$<br>$t_{13} = +0.76$<br>$p = 0.46$
Attention	$\beta = +0.022$<br>$SE = 0.12$<br>$t_{13} = +0.18$<br>$p = 0.86$	$\beta = +0.32$<br>$SE = 0.233$<br>$t_{13} = +1.38$<br>$p = 0.19$
Fluency	$\beta = -0.0834$<br>$SE = 0.0971$<br>$t_{13} = -0.86$<br>$p = 0.41$	$\beta = +0.096$<br>$SE = 0.188$<br>$t_{13} = +0.51$<br>$p = 0.62$
Language	$\beta = +0.0437$<br>$SE = 0.301$<br>$t_{13} = +0.15$<br>$p = 0.89$	$\beta = -0.41$<br>$SE = 0.584$<br>$t_{13} = -0.70$<br>$p = 0.50$
Memory	$\beta = +0.133$<br>$SE = 0.0737$<br>$t_{13} = +1.81$<br>$p = 0.09$	$\beta = +0.0312$<br>$SE = 0.143$<br>$t_{13} = +0.22$<br>$p = 0.83$
VisuoSpatial	$\beta = +0.156$<br>$SE = 0.496$<br>$t_{13} = +0.31$<br>$p = 0.76$	$\beta = -0.337$<br>$SE = 0.96$<br>$t_{13} = -0.35$<br>$p = 0.73$
$adj - R^2$	0.07	0.04
N_{obs}	19	19
AIC	54.02	63.63

Table S20: **Robust regression model investigating the correlation between the subdomains of Addenbrooke's Cognitive Examination (ACE III) and sensitivity to reward and uncertainty in ALE patients. Behavioural data is from Exp. 2.** Models were specified as follows: Sensitivity $\sim$ 1 + Attention + Memory + Fluency + Language + VisuoSpatial.

	Exp. 2	Exp. 3	Exp .4
(Intercept)	$\beta = -0.426$ $SE = 0.324$ $t_{3748} = -1.32$ $p = 0.19$	$\beta = +1.94$ $SE = 0.462$ $t_{4739} = +4.19$ ***p<0.0001***	$\beta = +2.19$ $SE = 0.618$ $t_{4184} = +3.54$ ***p = 0.0004***
ALE	$\beta = -0.403$ $SE = 0.459$ $t_{3748} = -0.88$ $p = 0.38$	$\beta = -0.131$ $SE = 0.644$ $t_{4739} = -0.20$ $p = 0.84$	$\beta = -1.01$ $SE = 0.991$ $t_{4184} = -1.02$ $p = 0.31$
ALE:Reward	$\beta = -0.984$ $SE = 0.275$ $t_{3748} = -3.58$ ***p = 0.00035***	$\beta = -0.466$ $SE = 0.362$ $t_{4739} = -1.29$ $p = 0.20$	$\beta = -1.84$ $SE = 0.583$ $t_{4184} = -3.16$ ***p = 0.0016***
Reward	$\beta = +1.41$ $SE = 0.198$ $t_{3748} = +7.15$ ***p<0.0001***	$\beta = +2.92$ $SE = 0.268$ $t_{4739} = +10.90$ ***p<0.0001***	$\beta = +2.35$ $SE = 0.371$ $t_{4184} = +6.33$ ***p<0.0001***
ALE:Effort		$\beta = +0.29$ $SE = 0.476$ $t_{4739} = +0.61$ $p = 0.54$	$\beta = +2.06$ $SE = 0.554$ $t_{4184} = +3.71$ ***p = 0.00021***
ALE:Reward:Effort		$\beta = -0.237$ $SE = 0.412$ $t_{4739} = -0.58$ $p = 0.56$	$\beta = +0.115$ $SE = 0.194$ $t_{4184} = +0.59$ $p = 0.55$
Effort		$\beta = -2.79$ $SE = 0.345$ $t_{4739} = -8.07$ ***p<0.0001***	$\beta = -2.38$ $SE = 0.354$ $t_{4184} = -6.71$ ***p<0.0001***
Reward:Effort		$\beta = -0.2$ $SE = 0.301$ $t_{4739} = -0.67$ $p = 0.51$	$\beta = +0.0131$ $SE = 0.133$ $t_{4184} = +0.10$ $p = 0.92$
ALE:EE	$\beta = +0.333$ $SE = 0.443$ $t_{3748} = +0.75$ $p = 0.45$		
LE:Reward:EE	$\beta = +0.158$ $SE = 0.17$ $t_{3748} = +0.93$ $p = 0.35$		
EE	$\beta = -2.73$ $SE = 0.314$ $t_{3748} = -8.71$ ***p<0.0001***		
Reward:EE	$\beta = +0.0727$ $SE = 0.125$ $t_{3748} = +0.58$ $p = 0.56$		
ALE:Uncertainty			$\beta = +0.887$ $SE = 0.893$ $t_{4184} = +0.99$ $p = 0.32$
ALE:Uncertainty:Effort			$\beta = -0.539$ $SE = 0.274$ $t_{4184} = -1.97$ ***p = 0.049***
ALE:Uncertainty:Reward			$\beta = +0.684$ $SE = 0.288$ $t_{4184} = +2.37$ ***p = 0.018***
ALE:Uncertainty:Reward:Effort			$\beta = -0.168$ $SE = 0.244$ $t_{4184} = -0.69$ $p = 0.49$
Uncertainty			$\beta = -1.82$ $SE = 0.556$ $t_{4184} = -3.27$ ***p = 0.0011***
Uncertainty:Effort			$\beta = +0.556$ $SE = 0.19$ $t_{4184} = +2.92$ ***p = 0.0035***
Uncertainty:Reward			$\beta = -0.852$ $SE = 0.196$ $t_{4184} = -4.35$ ***p<0.0001***
Uncertainty:Reward:Effort			$\beta = +0.12$ $SE = 0.167$ $t_{4184} = +0.72$ $p = 0.47$
$adj - R^2$	0.93	0.98	0.98
N_{obs}	3756	4747	4200
AIC	2940.79	2764.36	3342.31

Table S21: **Generalised mixed-effects model of the effect of the group (LE vs. controls) in Exp. 2-4 with per-trial random effect.** Models were specified as explained in Tables S8,S10 & S14 with the addition of (1 |trial).

	Exp. 2	Exp. 3	Exp .4
(Intercept)	$\beta = -0.595$ $SE = 0.355$ $t_{1848} = -1.68$ $p = 0.09$	$\beta = +2.23$ $SE = 0.513$ $t_{2364} = +4.35$ ***p<0.0001***	$\beta = +0.634$ $SE = 0.733$ $t_{1592} = +0.86$ $p = 0.39$
Task Half	$\beta = -0.638$ $SE = 0.195$ $t_{1848} = -3.28$ ***p = 0.0011***	$\beta = +0.0516$ $SE = 0.356$ $t_{2364} = +0.14$ $p = 0.88$	$\beta = -0.0312$ $SE = 0.194$ $t_{1592} = -0.16$ $p = 0.87$
Task Half:Reward	$\beta = +0.212$ $SE = 0.182$ $t_{1848} = +1.16$ $p = 0.25$	$\beta = +0.356$ $SE = 0.255$ $t_{2364} = +1.39$ $p = 0.16$	$\beta = -0.0395$ $SE = 0.159$ $t_{1592} = -0.25$ $p = 0.80$
Reward	$\beta = +0.384$ $SE = 0.175$ $t_{1848} = +2.19$ ***p = 0.029***	$\beta = +2.64$ $SE = 0.303$ $t_{2364} = +8.72$ ***p<0.0001***	$\beta = +0.425$ $SE = 0.218$ $t_{1592} = +1.94$ $p = 0.05$
Task Half:Effort		$\beta = -0.975$ $SE = 0.265$ $t_{2364} = -3.68$ ***p = 0.00024***	$\beta = +0.311$ $SE = 0.204$ $t_{1592} = +1.53$ $p = 0.13$
Task Half:Reward:Effort		$\beta = -0.731$ $SE = 0.275$ $t_{2364} = -2.66$ ***p = 0.0079***	$\beta = +0.0846$ $SE = 0.217$ $t_{1592} = +0.39$ $p = 0.70$
Effort		$\beta = -2.48$ $SE = 0.456$ $t_{2364} = -5.44$ ***p<0.0001***	$\beta = -0.464$ $SE = 0.263$ $t_{1592} = -1.76$ $p = 0.08$
Reward:Effort		$\beta = -0.236$ $SE = 0.317$ $t_{2364} = -0.74$ $p = 0.46$	$\beta = -0.0302$ $SE = 0.177$ $t_{1592} = -0.17$ $p = 0.86$
Task Half:EE	$\beta = -0.717$ $SE = 0.317$ $t_{1848} = -2.26$ ***p = 0.024***		
Task Half:Reward:EE	$\beta = -0.0899$ $SE = 0.228$ $t_{1848} = -0.39$ $p = 0.69$		
EE	$\beta = -2.22$ $SE = 0.311$ $t_{1848} = -7.13$ ***p<0.0001***		
Reward:EE	$\beta = +0.39$ $SE = 0.158$ $t_{1848} = +2.47$ ***p = 0.014***		
$adj - R^2$	0.91	0.90	0.75
N_{obs}	1856	2372	1600
AIC	1571.64	1468.39	1659.40

Table S22: **Generalised mixed-effects model of the effect of the Task Half (fist half vs second) on acceptance in Exp. 2–4.** Models were specified as follows. Exp. 2: choice $\sim$ 1 + Task Half*Reward + Task Half*EE + Reward*EE + Task Half:Reward:EEbest + (1 + Task Half*Reward + Task Half*EE + Reward*EE + Task Half:Reward:EE |Participant) + (1 |trial); Exp. 3: choice $\sim$ 1 + Task Half*Reward + Task Half*Effort + Reward*Effort + Task Half:Reward:Effort + (1 + Task Half*Reward + Task Half*Effort + Reward*Effort + Task Half:Reward:Effort |Participant) + (1 |trial); Exp .4: choice $\sim$ 1 + Task Half*Reward + Task Half*Effort + Reward*Effort + Task Half:Reward:Effort + (1 + Task Half*Reward + Task Half*Effort + Reward*Effort + Task Half:Reward:Effort |Participant) + (1 |trial).

	Catch Trials (Uncertainty Estimation)	Catch Trials (Decisions)
(Intercept)	$\beta = -0.692$<br>$SE = 0.0451$<br>$t_{206} = -15.35$<br>***p<0.0001***	$\beta = +2.55$<br>$SE = 2.83$<br>$t_{201} = +0.90$<br>$p = 0.37$
$Uncertainty_{High}$	$\beta = +0.463$<br>$SE = 0.0712$<br>$t_{206} = +6.49$<br>***p<0.0001***	$\beta = -7.31$<br>$SE = 3.61$<br>$t_{201} = -2.03$<br>***p = 0.044***
ALE	$\beta = +0.0428$<br>$SE = 0.0729$<br>$t_{206} = +0.59$<br>$p = 0.56$	$\beta = +3.42$<br>$SE = 3.88$<br>$t_{201} = +0.88$<br>$p = 0.38$
ALE:$Uncertainty_{High}$	$\beta = +0.00569$<br>$SE = 0.115$<br>$t_{206} = +0.05$<br>$p = 0.96$	$\beta = -0.914$<br>$SE = 4.53$<br>$t_{201} = -0.20$<br>$p = 0.84$
$Uncertainty_{High}$:Effort		$\beta = -3.48$<br>$SE = 2.32$<br>$t_{201} = -1.50$<br>$p = 0.13$
ALE:Effort		$\beta = +6.63$<br>$SE = 2.42$<br>$t_{201} = +2.74$<br>***p = 0.0068***
ALE:Reward		$\beta = -2.67$<br>$SE = 1.83$<br>$t_{201} = -1.46$<br>$p = 0.15$
Effort		$\beta = -3.75$<br>$SE = 2.27$<br>$t_{201} = -1.65$<br>$p = 0.10$
Reward		$\beta = +4.57$<br>$SE = 1.75$<br>$t_{201} = +2.61$<br>***p = 0.0098***
$adj - R^2$	0.89	1.00
N_{obs}	210	210
AIC	-189.27	182.40

Table S23: **Generalised mixed-effect model investigating group differences in catch trials (Exp. 4).** Models were specified as follows. Catch Trials (Uncertainty Estimation): Subjective Uncertainty (raw score) $\sim$ 1 + group*Uncertainty + (1 + Uncertainty |participant) + (1 |trial); Catch Trials (Decisions): choice $\sim$ 1 + group*Uncertainty + group*Reward + group*Effort + Uncertainty*Effort + (1 + Uncertainty*Effort |participant). Control group and low uncertainty level were set as references for group and uncertainty variables.

	Decision Time Exps. 2–4
(Intercept)	$\beta = +2.1$<br>$SE = 0.116$<br>$t_{91} = +18.22$<br>***p<0.0001***
Exp.3	$\beta = +0.128$<br>$SE = 0.136$<br>$t_{91} = +0.94$<br>$p = 0.35$
Exp.3:ALE	$\beta = +0.225$<br>$SE = 0.192$<br>$t_{91} = +1.17$<br>$p = 0.25$
Exp.4	$\beta = +1.18$<br>$SE = 0.154$<br>$t_{91} = +7.68$<br>***p<0.0001***
Exp.4:ALE	$\beta = -0.311$<br>$SE = 0.241$<br>$t_{91} = -1.29$<br>$p = 0.20$
ALE	$\beta = -0.057$<br>$SE = 0.163$<br>$t_{91} = -0.35$<br>$p = 0.73$
$adj - R^2$	0.60
N_{obs}	97
AIC	151.12

Table S24: **Generalised mixed-effects models investigating decision times across Exps. 2–3.** Models were specified as follows. Decision Time: DT $\sim$ 1 + Exp*group + (1 |Participant). Exp. 2 was set as the reference Exp.

	Exp. 1 (cds)		Exp. 2 (cds)		Exp. 3 (apples)		Exp. 4 (cds)	
**Group**	**Controls**	**ALE**	**Controls**	**ALE**	**Controls**	**ALE**	**Controls**	**ALE**
**Mean Total Score**	3941.87	3466.06	595.11	555.9	53.56	51.11	932.64	693.68
**SD**	211.14	483.42	106.33	141.72	9.02	9.26	155.97	387.51
**p-value**	< 0.001**		0.55		0.08		0.42	
**Reward in £***	£1 per 150 cds				£1 per 10 apples		£1 per 150 cds	

Table S25: **Scores in Exps. 1–4.*** While participants were told that this is the reward structure of the tasks, most of them were paid a maximum of £5 per experiment. Similar to Exp. 2, participants were paid for 1/10 of the trials in Exp. 1. ** Please refer to Table S4 for more details about the effect of different conditions on scores in Exp. 1. Group difference was investigated using a two-sample t-test if data fulfilled parametric assumptions or a Wilcoxon rank-sum test if assumptions were violated.

References

[1] Pierre Petitet, Bahaaeddin Attaallah, Sanjay G Manohar, and Masud Husain. The computational cost of active information sampling before decision-making under uncertainty. Nature Human Behaviour, pages 1–12, 5 2021.

[2] Bahaaeddin Attaallah, Pierre Petitet, Elista Slavkova, Vicky Turner, Youssuf Saleh, Sanjay G. Manohar, and Masud Husain. Hyperreactivity to uncertainty is a key feature of subjective cognitive impairment. eLife, 11, 5 2022.

[3] Luigi Acerbi and Wei Ji Ma. Practical Bayesian optimization for model fitting with Bayesian adaptive direct search. In Advances in Neural Information Processing Systems, volume 2017-Decem, pages 1837–1847, 2017.

[4] Lisa Nobis, Sanjay G. Manohar, Stephen M. Smith, Fidel Alfaro-Almagro, Mark Jenkinson, Clare E. Mackay, and Masud Husain. Hippocampal volume across age: Nomograms derived from over 19,700 people in UK Biobank. NeuroImage: Clinical, 23:101904, 1 2019.
