# Supplementary material for: The role of the human hippocampus in decision-making under uncertainty
Source: Nat Hum Behav. 2024 Apr 29;8(7):1366–82. doi: 10.1038/s41562-024-01855-2 (PMC11272595; doi:10.1038/s41562-024-01855-2)
Supplement: Supplementary file 2 — Reporting Summary [file 41562_2024_1855_MOESM2_ESM.pdf]

nature portfolio

Corresponding author(s): Bahaaeddin Attaallah

Last updated by author(s): 19-02-2024

Reporting Summary

Nature Portfolio wishes to improve the reproducibility of the work that we publish. This form provides structure for consistency and transparency in reporting. For further information on Nature Portfolio policies, see our Editorial Policies and the Editorial Policy Checklist.

Statistics

For all statistical analyses, confirm that the following items are present in the figure legend, table legend, main text, or Methods section.

n/a	Confirmed	
☐	☒	The exact sample size (*n*) for each experimental group/condition, given as a discrete number and unit of measurement
☐	☒	A statement on whether measurements were taken from distinct samples or whether the same sample was measured repeatedly
☐	☒	The statistical test(s) used AND whether they are one- or two-sided<br>*Only common tests should be described solely by name; describe more complex techniques in the Methods section.*
☐	☒	A description of all covariates tested
☐	☒	A description of any assumptions or corrections, such as tests of normality and adjustment for multiple comparisons
☐	☒	A full description of the statistical parameters including central tendency (e.g. means) or other basic estimates (e.g. regression coefficient) AND variation (e.g. standard deviation) or associated estimates of uncertainty (e.g. confidence intervals)
☐	☒	For null hypothesis testing, the test statistic (e.g. *F*, *t*, *r*) with confidence intervals, effect sizes, degrees of freedom and *P* value noted<br>*Give P values as exact values whenever suitable.*
☐	☒	For Bayesian analysis, information on the choice of priors and Markov chain Monte Carlo settings
☐	☒	For hierarchical and complex designs, identification of the appropriate level for tests and full reporting of outcomes
☐	☒	Estimates of effect sizes (e.g. Cohen's *d*, Pearson's *r*), indicating how they were calculated

Our web collection on statistics for biologists contains articles on many of the points above.

Software and code

Policy information about availability of computer code

Data collection	MATLAB (The MathWorks inc., version 2018b), Psychtoolbox v3.
Data analysis	MATLAB (The MathWorks inc., version 2019a), R version 3.5.2, Freesurfer version 7.1, FSL version 6.0.

For manuscripts utilizing custom algorithms or software that are central to the research but not yet described in published literature, software must be made available to editors and reviewers. We strongly encourage code deposition in a community repository (e.g. GitHub). See the Nature Portfolio guidelines for submitting code & software for further information.

Data

Policy information about availability of data

All manuscripts must include a data availability statement. This statement should provide the following information, where applicable:

- Accession codes, unique identifiers, or web links for publicly available datasets
- A description of any restrictions on data availability
- For clinical datasets or third party data, please ensure that the statement adheres to our policy

Anonymised participant data have been deposited on the Open Science Framework platform: https://osf.io/u4n2a/.

Research involving human participants, their data, or biological material

Policy information about studies with human participants or human data. See also policy information about sex, gender (identity/presentation), and sexual orientation and race, ethnicity and racism.

Reporting on sex and gender	Gender was determined based on self-reports.<br>Used only for purposes of matching patient and controls and as covariate where applicable.
Reporting on race, ethnicity, or other socially relevant groupings	N/A
Population characteristics	Exps. 1, 2 and 3:<br>N = 36 (19 ALE patients and 19 healthy Controls).<br>Age and gender: ALE (age: µ = 60.00, SD = ±11.36, 13 males). Controls (age: µ = 61.16, SD = ±11.71, 13 males).<br><br>In Exp. 4, N = 20 (eight ALE patients and 12 controls)<br>Age and gender: ALE (age: µ =55, SD = ±12.55, 6males). Controls (µ = 64.76, SD = ±7.42, 9 males)<br><br>Details characteristics of the two groups are described Tables in S1, S2, and S3 in Supplementary materials.
Recruitment	The ALE patient group was recruited based on a confirmed diagnosis through direct referrals from clinicians, whenever a patient with the condition was identified, in addition to the pre-existing pool of patients known to the cognitive neurology group in Oxford. All eligible patients identified during the data collection period were contacted and recruited upon their agreement to participate in the study. Age- and gender-matched controls were selected from a pool of volunteers who expressed interest in contributing to cognitive neurology research in Oxford. A computer code that matched age and gender and randomly selected eligible controls was utilized. Contact with potential candidates was established via phone and/or email.
Ethics oversight	University of Oxford ethics committee (RAS ID: 248379, Ethics Approval Reference: 18/SC/0448)

Note that full information on the approval of the study protocol must also be provided in the manuscript.

Field-specific reporting

Please select the one below that is the best fit for your research. If you are not sure, read the appropriate sections before making your selection.

☐ Life sciences ☒ Behavioural & social sciences ☐ Ecological, evolutionary & environmental sciences

For a reference copy of the document with all sections, see nature.com/documents/nr-reporting-summary-flat.pdf

Behavioural & social sciences study design

All studies must disclose on these points even when the disclosure is negative.

Study description	Quantitative case control lesion study. Data mainly from behavioural experiments.
Research sample	Autoimmune limbic encephalitis patients with LGI1/CASPRE2 anti-bodies and age- and gender matched controls. Demographics and group characteristics are summarized in Tables S1 and S2 of the manuscript. 19 individuals with a previously established diagnosis of ALE (age: µ = 60.00, SD = ±11.36, 13 males) were tested along with 19 healthy age- and gender-matched controls (age: µ = 61.16, SD = ±11.71, 13 males). In Exp. 4, eight ALE patients and 12 controls completed an additional follow-up task.<br><br>ALE patients are considered a lesion model for for focal hippocampal damage in humans, which is key to the question of the study investigating the role of the hippocampus in decision making. The study sample is representative of this group of patients with confirmed diagnosis and neuroimaging findings showing hippocampal atrophy.
Sampling strategy	Sampling relied on convenience and availability of patients as they present to the neurology clinic in Oxford or if they known patients from our database agreed to take part. Given the rarity of the condition, any patient in our data base who fitted the inclusion criteria was referred to take part in the study when it was running or invited to participate. Sample size was determined based on previous comparable work in ALE patients (Hanert et al., 2019; Spano et al., 2020a,b) as well as `previous research using the behavioural paradigms used in the study (Attaallah et al., 2022; Le Heron et al., 2018a,b; Petitet et al., 2021). See also description of recruitment above.
Data collection	All tasks were presented on a 17-inch touchscreen PC using MATLAB version 2018a and Psychtoolbox version 3. Participants sat in a quiet testing room, within reaching distance of the screen (about 50 cm). Questionnaire data were collected on an iPad tablet using RedCaP and Qualtrics.<br><br>An experimenter (sitting about 2-3 meters behind the participants) was present in the room at all time during behavioural testing. Their role was to explain the task at the beginning of the session (using an automated instruction script), and check that participants

were engaged in the task throughout the session. They did not provide strategy advices when participants asked.

Researchers were not blind to the experimental condition or study hypothesis, but their influence on performance was abolished by the use of automated, computerised testing procedures.

MRI scans were obtained at Acute Vascular Imaging Centre (AVIC) at John Radcliff Hospital (Oxford) using SIEMENS Verio 3T scanner. Detailed description of the data collection process is explained in the manuscript.

Timing	Experiments 1--3: Feb 2019- Feb 2020.<br>Experiment 4: Feb 2022 May 2022 (Post-covid interruption, especially collecting data from vulnerable patient group)
Data exclusions	No exclusions.<br>A number of rials from one of the patients for Exp. 2 were lost due to technical error. Data was still usable.
Non-participation	Exp.4:<br>11 patients: death (1), clinical deterioration (2), moved abroad (1), did not reply or declined (7).<br>7 controls: moved abroad (1), did not reply or declined (6).
Randomization	Participants were assigned to two groups: controls and cases. The cases were ALE patients. No randomisation is applicable across the two groups.<br>However, behavioural task trials were randomised and study blocks were counter-balanced across participants.

Reporting for specific materials, systems and methods

We require information from authors about some types of materials, experimental systems and methods used in many studies. Here, indicate whether each material, system or method listed is relevant to your study. If you are not sure if a list item applies to your research, read the appropriate section before selecting a response.

Materials & experimental systems

n/a	Involved in the study	
☒	☐	Antibodies
☒	☐	Eukaryotic cell lines
☒	☐	Palaeontology and archaeology
☒	☐	Animals and other organisms
☒	☐	Clinical data
☒	☐	Dual use research of concern
☒	☐	Plants

Methods

n/a	Involved in the study	
☒	☐	ChIP-seq
☒	☐	Flow cytometry
☐	☒	MRI-based neuroimaging

Magnetic resonance imaging

Experimental design

Design type	N/A: Offline structural imaging.
Design specifications	N/A: no task-fMRI data collected
Behavioral performance measures	N/A: Reward and uncertainty sensitivity from Exp. 2 from offline behavioural tasks (not task-fMRI).

Acquisition

Imaging type(s)	Structural
Field strength	3T
Sequence & imaging parameters	r. High-resolution T1-weighted structural MR images (MPRAGE; 208 sagittal slices of 1 mm thickness, voxel size = 1 mm isotropic, TR/TE = 2000/1.94 ms; flip angle = 8∘, FOV read = 256, iPAT =2, prescan-normalise) and T2 weighted fluid attenuated inversion recovery (FLAIR) images (192 sagittal slices of 1.05 mm thickness, voxel size = 1×1×1.1, TR/TE = 5000/397 ms; FOV read = 256; iPAT = 2, partial Fourier = 7/8, fat saturation, prescan-normalise) were acquired.
Area of acquisition	Whole brain.

Diffusion MRI ☐ Used ☒ Not used

Preprocessing

Preprocessing software	FSL 6.0 and FreeSurfer 7.1.

Normalization	Non-linear registration was used to register structural brain-extracted and grey matter-segmented images to the MNI 152 standard space.
Normalization template	MNI 152
Noise and artifact removal	N/A: Structural imaging
Volume censoring	N/A: Structural imaging

Statistical modeling & inference

Model type and settings	N/A: Structural analysis with GLM investigating VBM group differences.
Effect(s) tested	N/A

Specify type of analysis: ☒ Whole brain ☐ ROI-based ☐ Both

Statistic type for inference (See Eklund et al. 2016)	VBM (voxel-wise).
Correction	Non-parametric testing using randomise in FSL with 5000 permutations and corrected for multiple comparisons across space was used to detect voxel-wise differences in grey matter volumes between patients and controls.

Models & analysis

n/a	Involved in the study
☒	☐ Functional and/or effective connectivity
☒	☐ Graph analysis
☒	☐ Multivariate modeling or predictive analysis
